# Computational neural modelling of auditory cortical receptive fields

**DOI:** 10.1186/1471-2202-16-S1-P85

**Published:** 2015-12-18

**Authors:** Jordan D Chambers, Anthony N Burkitt, David B Grayden

**Affiliations:** 1 NeuroEngineering Laboratory, Department of Electrical and Electronic Engineering, University of Melbourne, Parkville, Victoria 3010, Australia; 2 Bionics Institute, East Melbourne, Victoria 3002, Australia; 3 Centre for Neural Engineering, University of Melbourne, Parkville, Victoria 3010, Australia

## Introduction

Acoustic signals are mainly characterized by their temporal dynamics. Electrophysiological studies have shown that neurons in the primary auditory cortex (A1) can detect fine temporal structure of acoustic stimuli [1]. The traditional view of auditory processing describes how the temporal codes of sounds are distributed in the frequency domain along the auditory pathway from the basilar membrane to the cortex. Therefore, it is important to consider a sound's spectral and temporal features together. The spectrotemporal receptive field (STRF) is a description of the auditory system's input-to-output transformation encompassing both the spectral and temporal features. The STRF of A1 neurons exhibit complex patterns that can undergo rapid task-related changes [2]. However, the mechanisms by which cortical neurons change their STRF remains unclear.

## Methods

A computational neural model was developed to investigate mechanisms by which cortical neurons can change their STRF. A sound signal is played into a model of the cochlear and cochlear nucleus (CN) [3]. It comprises of a bank constant-Q bandpass filters spread along the frequency axis, a high-pass filter, a non-linear compression, a low-pass filter, a first-order derivative with respect to the tonotopic axis, a half-wave rectifier and integration over a 8 ms window. The output of the CN tuned to a particular frequency drives the input to integrate-and-fire neuron models to represent cortical neurons. Action potentials (AP) in the cortical neurons are then used to calculate a reverse autocorrelation of the STRF.

## Results

When white Gaussian noise is used as the sound signal, a region of STRF excitation is produced with a frequency corresponding to the CN output. This STRF excitatory region can be increased in the frequency domain by increasing the number of CN outputs exciting the cortical neuron. If there is a large separation along the frequency axis, multiple STRF excitatory regions are created. The excitatory region can be altered in the temporal domain by adding delays to the CN output. Regions of STRF inhibition can also be produced when a cortical neuron fires APs tonically and the CN output hyperpolarizes the cortical neuron. Alternatively, a region of STRF inhibition can be created without tonic firing, if one CN output excites the cortical neuron and a different CN output hyperpolarizes the cortical neuron. This creates a region of excitation and a region of inhibition in the STRF, similar to complex STRFs observed experimentally. STRF inhibitory regions can be altered in the frequency and temporal domains in the same way as excitatory regions. Changing the properties of the cortical neuron to include an after-hyperpolarizing potential following an AP causes a region of excitation to be preceded by a region of inhibition, and a region of inhibition to be preceded by a region of excitation, hereby providing a secondary means to producing complex STRFs.

## Conclusions

This computational neural model of STRF in A1 can reproduce experimentally observed complex STRFs. It can predict changes in synaptic connectivity and AP firing properties occurring in A1 neurons corresponding to rapid task-dependent changes in the STRFs.
